# The Odor of Musk Destroyed by Camphor

**Published:** 1851-01

**Authors:** 


*The Odor of Musk destroyed by Camphor.*—The combination of camphor with musk, according to M. Fleischman, destroys the odor of the latter.

